# Comprehensive Modeling of Acetone Clusters: QTAIM Analysis and QCE Study

**DOI:** 10.1002/jcc.70380

**Published:** 2026-04-20

**Authors:** Juda Baikété, Alhadji Malloum, Jeanet Conradie

**Affiliations:** ^1^ Department of Physics, Faculty of Science University of Maroua Maroua Cameroon; ^2^ Department of Chemistry University of the Free State Bloemfontein South Africa

**Keywords:** acetone clusters, heat capacity, IR, QCE theory, QTAIM analysis

## Abstract

In molecular research, comprehending the microscopic source of the macroscopic characteristics of polar aprotic solvents continues to be a significant difficulty. In order to bridge the gap between cluster‐scale interactions and liquid acetone properties, we present a thorough quantum‐chemical and statistical modeling of neutral acetone clusters in this work. The ABCluster algorithm was used to thoroughly explore the potential energy surface. High‐level density functional theory calculations at the MN12SX‐D3/def2‐TZVP level were then performed, benchmarked against DLPNO‐CCSD(T)/CBS reference energies. A thorough Quantum Theory of Atoms in Molecules (QTAIM) analysis of the nature and hierarchy of intermolecular interactions revealed a cooperative network dominated by dipole‐dipole O⋯C and O⋯O interactions, supplemented by numerous weak C‐H⋯O, H⋯C, and H⋯H dispersive contacts. The application of the QCE theory predicts a distribution dominated by trimers at low temperatures (T< 200 K), leading to a predominance of monomers above 260–280 K, reflecting the subtle equilibrium between electrostatic stabilization and entropic effects. The model reproduces experimental thermodynamic properties, such as the thermal capacity (Cp) between 200 and 375 K and infrared spectra at 300 K, with the calculated band of elongation C=O (1710 cm^–1^) being just 5 cm^–1^ from the experimental value (1715 cm^–1^). The thermodynamic properties and infrared spectrum of liquid acetone predicted by QCE show excellent agreement with experimental data, thus validating the integrated DFT‐QTAIM‐QCE approach. This work provides the first complete QCE characterization of pure liquid acetone, demonstrating that its macroscopic properties emerge from a dynamic equilibrium of small, weakly‐bound clusters rather than extended hydrogen‐bonded networks, and establishes a validated computational framework for predicting liquid‐phase properties from ab initio cluster data.

## Graphical abstract

A multiscale DFT‐QTAIM‐QCE approach links acetone cluster interactions to liquid‐phase behavior. Macroscopic thermodynamic and IR properties emerge from a dynamic equilibrium of small clusters.

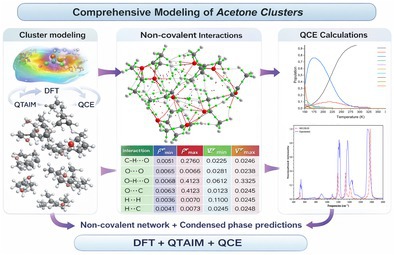

## 1 Introduction

Molecular clusters play a crucial role in understanding the thermodynamic and spectroscopic properties of the condensed phase. In this context, acetone aggregates are of particular interest due to the numerous applications of this molecule in fields such as atmospheric chemistry, organic solvents, liquid dynamics, the development of new spectroscopic methods for vapor detection, and the study of noncovalent interactions in organic media. Understanding the structure, stability, thermodynamics, and dynamics of these aggregates is essential for these fields.

The study of acetone clusters is thus situated within a rich scientific landscape where advanced experimental investigations and sophisticated theoretical modeling intersect. Although early structural studies using microwave spectroscopy focused on model systems such as water dimers [1], the methodology they established paved the way for the analysis of more complex organic molecules. For acetone, infrared spectroscopic techniques have proven crucial for probing its molecular environment, particularly in condensed phases. The work of Max and Chapados [2] on the IR spectroscopy of acetone‐water liquid mixtures provided detailed molecular models and highlighted the specific interactions, such as C‐H⋯O hydrogen bonds, that govern aggregation in this binary system. Jiwen et al. [3] used IR‐VUV spectroscopy for neutral clusters of acetone (n=1−4) and observed a shift in the C=O “overtone” band, going from ∼3455 to 3433 cm^−1^ when the number of monomers increases, which suggests that for the trimer and tetramer, the dominant structures are cyclic, stabilized by weak interactions between the carbonyl group and methyl groups. Furthermore, Xu et al. [4] in 2004 calculated the structures and vibrational spectra of protonated acetone clusters up to n=7 ${\left({\text{CH}}_{3}{\text{COCH}}_{3}\right)}_{\text{n}}{\text{H}}^{+}$ via Density Functional Theory (DFT) (B3LYP) and showed that with increasing cluster size, the proton affinity increases, and that the spectra exhibit a splitting of the C=O bond band as well as a weakening of the intensities, indicative of increasing proton solvation. Douberly et al. [5] experimentally obtained the IR spectra of the protonated monomer and the protonated dimer of acetone, confirming the vibrations predicted by ab initio calculations (MP2, low harmonics) and highlighting the nature of the proton‐bridging bonds in these aggregates. Thus, this research highlights the existence of tangible data relating to the structure, energy, and spectroscopy of neutral (up to n=4) and protonated (up to n=2 or more) acetone clusters. However, this data is limited either by its size or by its nature (neutral versus protonated).

At the same time, the evolution of quantum computing techniques has paved the way for increasingly precise exploration of the structure and energy of acetone clusters. Ab initio methods (such as MP2 and CCSD(T)) as well as DFT have been used to establish the geometries, binding energies, and vibrational frequencies of clusters, ranging from dimers to larger ensembles [6, 7]. These calculations have corroborated the type of interactions that predominate and have facilitated the prediction of the comparative stability of various structural isomers [8].

Weinhold [9] and Ludwig, then Weinhold and Farrar [10, 11] developed the quantum cluster equilibrium (QCE) model, which characterizes a liquid as a dynamic combination of clusters of different sizes whose macroscopic properties arise from the individual contributions of these aggregates. This statistical framework's adaptability as a predictive tool for thermodynamic and spectroscopic features has been demonstrated by its effective application to an increasing variety of pure liquids and binary mixes. Early uses concentrated on N‐methylacetamide [12] and liquid ammonia [10, 11], laying the methodological groundwork at the Hartree‐Fock level. The scope of QCE was expanded to methanol [13, 14], ethanol [15], and acetonitrile [16] in later studies using more advanced quantum chemical techniques. These studies consistently showed that the model could reproduce experimental thermodynamic properties and infrared spectra from ab initio cluster data alone. Kirchner and colleagues created the bQCE and multi‐QCE formalisms [17] for binary mixtures, which were successfully applied to water‐DMSO, water‐N‐methylformamide, and protic ionic liquids [18, 19], as well as methanol‐alcohol mixtures [20], hexafluoroisopropanol‐ethanol [21], and methanol‐chloroform [22]. These contributions validate the QCE approach's adaptability and resilience for first‐principles equilibrium property prediction of multicomponent systems. Despite this wide range of applications, there is still a significant gap in the literature: QCE theory has never been used and verified for the pure acetone cluster system, as far as we are aware. The majority of the current theoretical work on acetone concentrates on small aggregates in mixed systems [2, 3, 4, 5], and it does not enable the deduction of the macroscopic properties of the pure fluid or a predictive description of the thermodynamic equilibrium distribution of an entire ensemble of competing clusters. Therefore, applying QCE to acetone presents a distinct scientific opportunity that builds on the tried‐and‐true techniques developed for comparable liquids and closes this gap with a thorough, verified computational framework.

A review of previous work reveals that a large number of investigations using QCE theory are documented in the literature. Most models employ simplifications to characterize inter‐cluster interactions, such as the use of empirical parameters or the selection of a coarse‐grained (or approximate) large‐scale method. Existing work on acetone, whether spectroscopic or theoretical, focuses predominantly on small aggregates in mixed systems. They do not allow for a predictive description of the thermodynamic equilibrium distribution of a complete ensemble of competing clusters, nor for the deduction of the macroscopic properties of the pure fluid. It is also worth noting that, although QCE now appears to be a robust and validated method for several liquids (ammonia, ethanol, acetonitrile, etc.), a notable gap persists: to our knowledge, QCE theory has never been applied and validated for the pure acetone cluster system. Therefore, applying QCE to acetone, building on the proven methodologies for other liquids (water, ammonia, ethanol, etc.), represents a scientific opportunity to fill this clearly identifiable gap: the absence of a comprehensive QCE model for pure acetone.

In this work, we aim to apply QCE theory systematically and comprehensively to the study of neutral acetone clusters, in order to link their microscopic properties to those of the macroscopic liquid. Consequently, our specific objectives are as follows: (i) explore the potential energy surface of acetone clusters from n=1 to 10 by combining systematic generation via ABCluster and high‐precision DFT calculations; (ii) characterize intermolecular interactions and relative stabilizations using quantum chemistry tools and QTAIM analysis; (iii) determine the infrared vibrational signature of liquid acetone by weighting the calculated spectra of individual clusters according to their QCE population at 300 K; (iv) apply the QCE formalism to deduce the equilibrium populations and thermodynamic contributions of each cluster in the condensed phase; and finally (v) predict the macroscopic properties of liquid acetone such as the enthalpy of vaporization, density, and free energy, then compare these values to existing experimental data. The entirety of these objectives aims to fill the notable gap in the literature by providing the first complete description of liquid acetone based on both reliable quantum data and a rigorous statistical model.

Building upon our first study dedicated to acetone clusters of size n=1−8, characterized by DFT and analyzed via QTAIM, this second work extends the exploration to larger aggregates and integrates a comprehensive thermodynamic modeling based on QCE theory. To ensure coherence between the two contributions, we begin with a brief recap of the major results previously obtained: optimized structures, dominant interactions, and relative stability, in order to establish a solid analytical foundation. We then present the n=9 and n=10 clusters, new to this study, detailing their most stable conformations, their assembly modes, and complementing this with a QTAIM analysis. Finally, the complete set of clusters from n=1 to 10 serves as the basis for applying QCE theory to predict the equilibrium populations, the thermodynamic properties of liquid acetone, and its IR spectroscopic signatures. This integrated DFT‐QTAIM‐QCE approach thus provides a comprehensive view connecting cluster structure to the macroscopic properties of the liquid. Applying QCE to acetone therefore constitutes a clearly defined scientific opportunity, albeit one that brings distinct problems that distinguish it from previously examined systems. Acetone is an aprotic polar solvent that completely lacks traditional hydrogen bond donors, in contrast to ammonia [10, 11] and ethanol [15], which are protic solvents whose liquid structure is controlled by strong, directional O‐H⋯O or N‐H⋯N hydrogen bonds. Instead, our QTAIM analysis [8] shows that its intermolecular cohesion depends on a subtle interplay of dispersive C‐H⋯H‐C and H⋯C forces, weak C‐H⋯O contacts, and dipole‐dipole O⋯C interactions. This nondirectional and cooperative interaction network produces an unusually flat potential energy surface, generating a very large number of quasi‐degenerate isomers for each cluster size up to twenty‐nine for the hexamer and thirty‐seven for the heptamer; a degree of conformational complexity markedly exceeding that reported for ammonia or ethanol clusters of comparable size. Additionally, unlike acetonitrile [16], whose strong dipole moment (3.92 D) imposes a relatively well‐defined local orientational order, acetone's moderate dipole moment (2.88 D) combined with the steric bulk of its methyl groups results in a highly dynamic liquid structure dominated by short‐range correlations incompatible with the formation of large, stable clusters [23, 24]. The absence of conventional hydrogen bonds, extreme conformational degeneracy, and a nondirectional interaction network make pure acetone a particularly challenging test case for the QCE framework.

## 2 Methodology

### 2.1 QCE Theory

The statistical framework of the quantum cluster equilibrium (QCE) theory allows for the description of an ensemble of clusters in thermodynamic equilibrium within a condensed system. This method has already been extensively described in the literature [10, 13, 25]. In this approach, a liquid is considered as a dynamic combination of clusters of various sizes, and its macroscopic properties result from the individual contributions of these clusters. The total partition function $Q_{\text{tot}}$ of a canonical ensemble containing different clusters of size i is given by Equation (1), where T is the temperature, V the volume of the system, $N_{i}$ the number of clusters of size i, and $q_{i}$ the partition function of an individual cluster. The classical thermodynamic relation between volume and pressure is given by Equation (2). Each partition function $q_{i}$ is factored into translational, rotational, vibrational, and electronic contributions. These internal partition functions for each cluster are given by Equations ((3), (4), (5)), where $I_{1}$, $I_{2}$, and $I_{3}$ represent the principal moments of inertia, σ denotes the rotational symmetry number, $\Theta_{v,i}$ refers to the characteristic temperature of the i‐th vibrational mode, and $\varepsilon_{0}^{\text{el}}$ corresponds to the zero‐point energy (ZPE) adjusted electronic energy. Equation (6) provides the probability of observing a cluster of size p relative to the monomer, where $\mu_{p}$ Equation (7) corresponds to the cluster stoichiometry and $q_{p}$ is an equilibrium factor derived from the equality of chemical potentials.

(1) $$Q_{\text{tot}}(N_{i},V,T)=\overset{N}{\underset{i=1}{\prod }}\frac{{\left[q_{i}^{tot}(V,T)\right]}^{N_{i}}}{N_{i}!}$$

(2) $$p=k_{\text{B}}T{\left(\frac{\partial \ln Q_{\text{tot}}}{\partial V}\right)}_{T,N_{i}}$$

(3) $$q_{i}^{\text{rot}}(T)=\frac{\pi^{1/2}}{\sigma }{\left(\frac{8\pi^{2}k_{B}T}{h^{2}}\right)}^{3/2}\sqrt{I_{1}I_{2}I_{3}}$$

(4) $$q_{i}^{\text{vib}}(T)=\underset{i}{\prod }\frac{1}{1-\exp\left(-\frac{\Theta_{v,i}}{T}\right)}$$

(5) $$q_{i}^{\text{el}}(T)=g_{0}\;\exp\left(-\frac{\varepsilon_{0}^{\text{el}}}{k_{B}T}\right)$$

(6) $$N_{p}=q_{p}{\left(\frac{q_{p}}{q_{1}}\right)}^{n_{p}}$$

(7) $$\mu_{p}=-k_{\text{B}}T\frac{\partial \ln q_{p}}{\partial n_{p}}$$

Two empirical parameters, the mean‐field parameter $a_{\text{mf}}$ and the excluded volume parameter $b_{\text{xv}}$, are incorporated to refine the description of inter‐cluster interactions in the condensed phase. The former adjusts the electronic contribution according to Equation (8), while the latter acts on the translational contribution Equation (9), where λ represents the total excluded volume Equation (10), and $V_{\text{excl}}$ corresponds to the de Broglie wavelength Equation (11).

(8) $$q_{i}^{\text{el}}(V,T)=\exp\left[-\frac{E_{\text{elec}}-p(i)\;a_{\text{mf}}}{k_{\text{B}}T}\right]$$

(9) $$q_{p}^{\text{trans}}=\frac{V-V_{\text{excl}}}{\lambda^{3}}$$

(10) $$\lambda =\frac{h}{\sqrt{2\pi mk_{\text{B}}T}}$$

(11) $$V_{\text{excl}}=b_{\text{xv}}\underset{p}{\sum }N_{p}V_{p}$$

When $a_{\text{mf}}$ and $b_{\text{xv}}$ are not optimized, the model reduces to a gas‐phase representation (QCE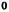). Using the experimental density and boiling point of acetone (ρ=0.7899 g.cm^−3^ at 298 K; T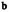 = 329.2 K) reported in the NIST Chemistry WebBook [26], the ideal values of these two parameters were found by minimizing the difference between QCE‐predicted and experimental liquid densities over the temperature range 200‐375 K. In the Peacemaker software, the optimization was carried out repeatedly until the isobar and the molar volume converged. The final ideal parameters are $b_{\text{xv}}$ = 1.7297 and $a_{\text{mf}}$ = 1.4064 J·m^3^ ·mol^−2^. These values are specific to the MN12SX‐D3/def2‐TZVP cluster data used as input and are not transferable to other levels of theory without re‐optimization. This work employs QCE theory through the Peacemaker [25, 27] software, which implements this formalism for both pure liquids and binary mixtures. It is important to note that the code requires, as input, the optimized Cartesian coordinates for each cluster, their binding energies, and their harmonic frequencies.

### 2.2 Computational Details

In our previous investigation on acetone clusters of size n=1 to 8, a progressive computational strategy was implemented to determine in detail the stable conformations and to estimate their structural and energetic properties [8]. The bee colony algorithm integrated into the ABCluster [28, 29] program was used for the initial exploration of the potential energy surfaces, employing the CHARMM force field to generate a wide range of plausible structural configurations. A subsequent in‐depth comparison phase helped identify the optimal theoretical level: twenty‐six dispersion‐corrected DFT‐D3 functionals were carefully benchmarked against highly accurate reference energies computed at the DLPNO‐CCSD(T)/CBS (Complete Basis Set) level. This study demonstrated the efficiency of the MN12SX‐D3 [30, 31] functional, combined with the polarized triple‐zeta def2‐TZVP basis set, in accurately describing the noncovalent interactions within these systems. All geometries were optimized and their vibrational frequencies were determined at this theoretical level. The gaussian 16 software package (Revision C.01, Gaussian Inc., Wallingford, CT, 2016) was used for all DFT geometry optimizations, vibrational frequency computations, and the ensuing QTAIM topological analyses [32]. For benchmarking, the high‐level DLPNO‐CCSD(T)/CBS reference energy calculations were performed using the orca program package (version 5.0.3, Max‐Planck‐Institut f'ur Kohlenforschung) [33]. The CHARMM force field as implemented in the abcluster program (version 2.1) [28] was used in the first conformational search using the ABCluster algorithm [28, 29]. The aimall software package (Version 19.10.12, TK Gristmill Software, Overland Park, KS, USA) [34] was used to perform topological analysis of the electron densities. The peacemaker software (version 2.8) [25, 27] was used to do the QCE computations. Binding energies $\left(\Delta E_{n}\right)$ were computed, and the relative populations of the various isomers at different temperatures were evaluated using the Boltzmann distribution. A total of 136 isomers were identified for n ranging from 2 to 8. To determine the globally most stable structures, ZPE‐corrected relative energies were calculated. Topological analysis of the electron densities was performed using QTAIM [35], enabling the identification of bond critical points (BCPs) and the characterization of C‐H⋯O, dipole‐dipole, and dispersive interactions. Trends in organization, compaction, and cooperative stabilization were analyzed as a function of cluster size. Candidate isomers were chosen for the thermodynamic population analysis based on the following criteria: (i) all isomers within an energy window of 1.5 kcal·mol^−1^ above the global minimum were systematically included, as they are expected to contribute significantly to the thermodynamic ensemble at low and moderate temperatures; (ii) higher‐energy isomers were additionally included if their Boltzmann population exceeded 5% at any temperature within the range 0‐500 K, as determined by a preliminary screening of all identified isomers; and (iii) isomers with distinct topological features (e.g., open‐chain vs. cyclic motifs) were kept to guarantee the ensemble's structural representativeness. This selection process ensures that the presented population distributions represent both the high‐temperature entropy‐driven regime of the cluster equilibrium and the low‐temperature energy‐driven regime. Additionally, clusters of size n=9 and 10 were calculated at the MN12SX‐D3/def2‐TZVP level.

## 3 Results and Discussion

### 3.1 Synthesis of Energy Trends, Structures and QTAIM Analysis of Acetone Aggregates n=2−8

Our previous contribution [8] reported and discussed in detail the structural and energetic results for acetone clusters of size n=2−8, including the optimized geometries, ZPE‐corrected relative energies, assembly topologies, and Boltzmann population analyses. In short, the optimization of van der Waals contacts and cooperative C‐H⋯O interactions drives the systematic evolution of the potential energy surface from almost planar, cyclic arrangements in small aggregates (n=2−4) toward compact three‐dimensional structures for larger (n=5−8) clusters. With up to twenty‐seven competing isomers found for the octamer within a small energy range of only 0.44 kcal·mol^−1^, the degree of conformational degeneracy increases dramatically with cluster size, confirming the essentially flat and nondirectional nature of the acetone intermolecular potential energy surface. The present work extends this exploration to the nonamer (n=9) and decamer (n=10), which represent the largest neutral acetone clusters characterized to date at this level of theory.

The QTAIM analysis of acetone clusters n=2−8, reported in detail in our previous work [8], identified four main types of noncovalent interactions based on their electron density ρ at the bonding critical point (BCP): dipole‐dipole O⋯C and O⋯O interactions, C‐H⋯O contacts, C‐H⋯π(C=O) interactions, and dispersive C‐H⋯H‐C and H⋯C forces. All interactions exhibit low electron density and positive Laplacian values, confirming their closed‐shell nature consistent with the polar but aprotic character of acetone. The final verdict was that the essentially complete electron shell nature of all these interactions, as indicated by the positive Laplacian values, perfectly coincides with the nonpolar yet polar nature of the acetone molecule. Furthermore, this agrees with previous spectroscopic research that associates the stabilization of the acetone clusters with dipole‐dipole and dispersion interactions rather than a true hydrogen‐bonding network [3, 36]. These results are extended here to the n=9 and n=10 clusters, as discussed in the following section.

### 3.2 Structures, Relative Energy and QTAIM Analysis for n=9 and n=10

***Structures***

**FIGURE 1:**
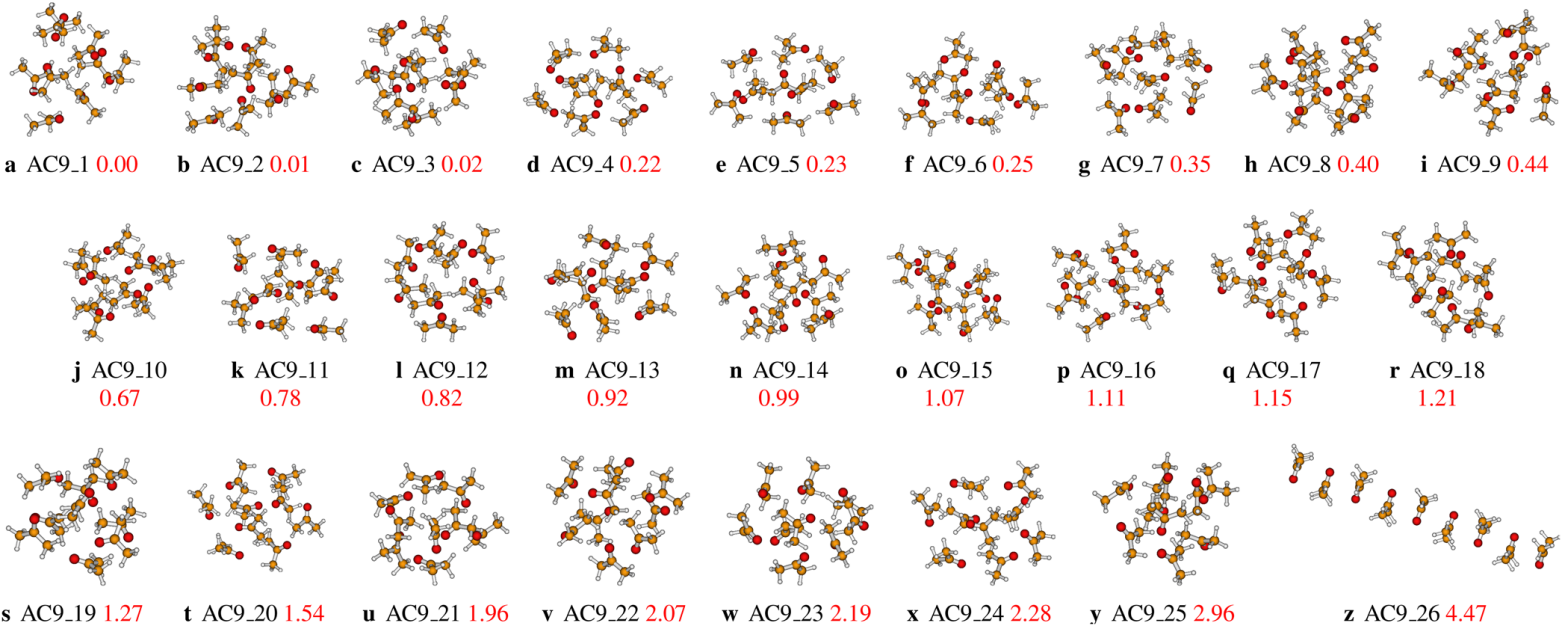
Different conformers of ${\left({\text{CH}}_{3}{\text{COCH}}_{3}\right)}_{\text{n}}$ clusters for n=9, optimized at the MN12SX‐D3/def2‐TZVP level of theory. The corrected relative zero‐point energy of the isomers, in kcal/mol, is represented by the numbers in red.

For the nonamer of acetone, twenty‐six stable isomers have been identified and reported in Figure 1 (optimized Cartesian coordinates of the isomers are provided in the Supporting Information). The most stable structure (AC9_1) adopts a compact three‐dimensional architecture, consistent with the trends noted for hexa, hepta, and octamers, confirming the systematic evolution toward 3D compactness with increasing cluster size. This observation coincides with previous research by Hu et al. [36], who emphasized the importance of cooperative effects and the nonlinear increase in binding energy per molecule as a function of the cluster diameter, as well as the work of Guan et al. [3], which indicates a simplification of IR spectra for larger aggregates, highlighting a rapid process of conformational averaging. The energy degeneracy is particularly notable, comprising nine isomers (AC9_1 to AC9_9) that lie within an extremely narrow energy range of less than 0.45 kcal/mol. The isomers AC9_2 and AC9_3 are almost isoenergetic to the global minimum, with an energy difference of +0.01 and +0.02 kcal/mol, respectively. This extreme reduction of the potential energy surface is maintained for a wide range of structures, with eighteen isomers located within a range of less than 1.3 kcal/mol. This amplified degradation supports the findings of Matsuda et al. [37] regarding the dynamic complexity of systems related to acetone, and echoes the observations of Faginas‐Lago et al. [23] on the plurality of accessible energy points even for medium‐sized aggregates. The arrangement of the elements is primarily characterized by compact 3D configurations, which aim to optimize the number of attractive van der Waals bonds and C‐H⋯O interactions. Only the isomers with the highest energy (AC9_25 at +2.96 kcal/mol and AC9_26 at +4.47 kcal/mol) are likely to be excluded from the ensemble at thermal equilibrium. These results confirm and amplify the conclusion drawn from smaller aggregates, in agreement with the studies of Malloum et al. [38, 39, 40, 41, 42] on organic solvent clusters: for clusters of this size, the condensed‐phase system must be considered as a dynamic and fluid mixture of a very large number of quasi‐degenerate conformational structures, rather than as an assembly dominated by a unique architecture. This perspective is consistent with the “soft” and nondirectional energy landscape model characteristic of interactions dominated by dispersion and dipoles, as described for acetone [3, 23, 36].

**FIGURE 2:**
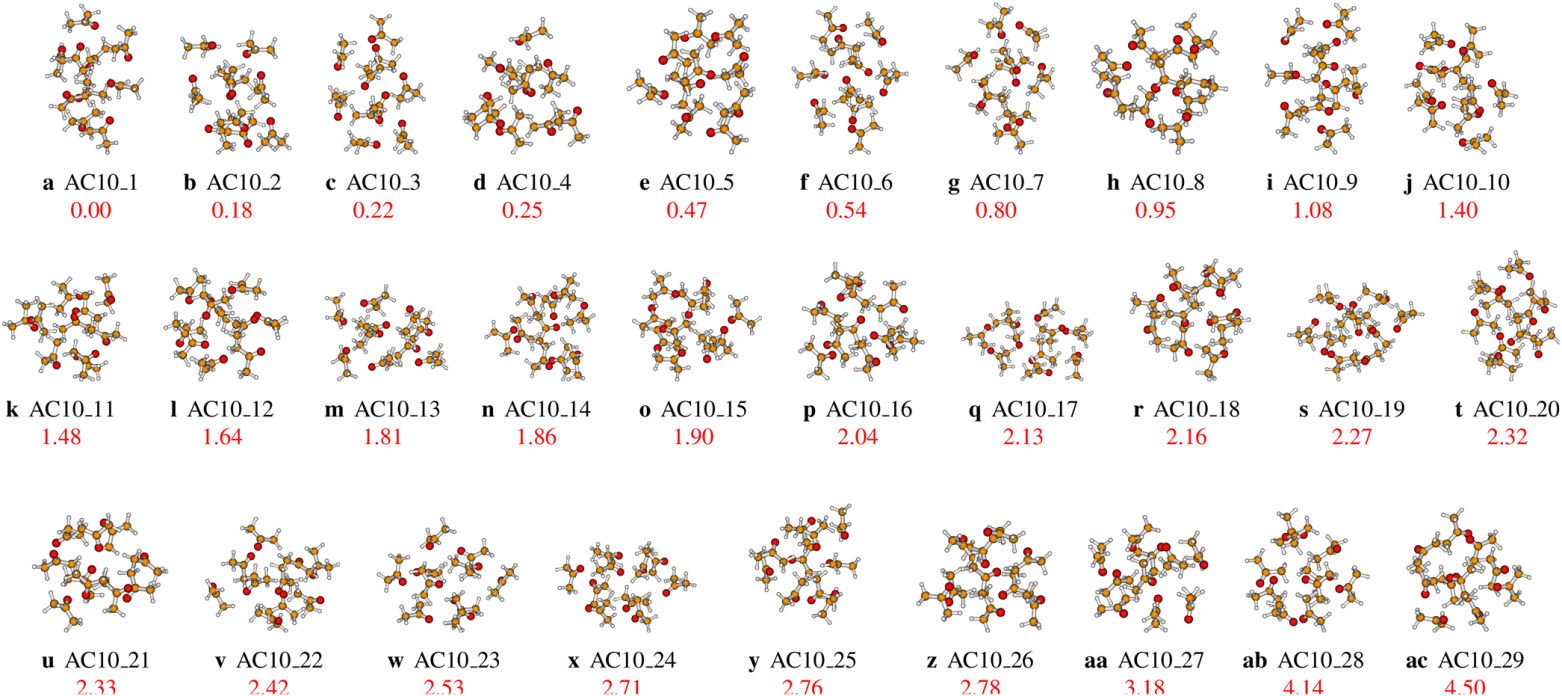
Different conformers of ${\left({\text{CH}}_{3}{\text{COCH}}_{3}\right)}_{\text{n}}$ clusters for n=10, optimized at the MN12SX‐D3/def2‐TZVP level of theory. The corrected relative zero‐point energy of the isomers, in kcal/mol, is represented by the numbers in red.

Twenty‐nine isomers were characterized and illustrated in Figure 2 for the acetone decamer, confirming the established trend for aggregates of size n ≥ 5 towards compact and energetically degenerate three‐dimensional architectures (optimized Cartesian coordinates of the isomers are provided in the Supporting Information). The global minimum (AC10_1) represents a compact structure designed to optimize both directional C‐H⋯O interactions and nondirectional dispersive forces, consistent with the stabilization mechanisms described by Aviyente and Vernali [43] and Hu et al. [36]. The observed quasi‐degeneracy process continues, including five isomers (AC10_1 to AC10_5) that lie within an energy range below 0.5 kcal/mol. Most of the structures (eighteen isomers) are located within a range of 1.6 kcal/mol. This property validates and extends the results of Faginas‐Lago et al. [23] concerning the multiple number of energy minima available in acetone aggregates, as well as the study by Guan et al. [3] which highlights the spectral “smoothing” linked to rapid conformational averaging in larger aggregates. The thermodynamic probability calculated by the Boltzmann distribution will necessarily indicate a population distributed over several states, without exclusive dominance of any particular conformation beyond cryogenic temperatures. This extreme conformational competition reflects the “soft” and nondirectional nature of the intermolecular potential of acetone, as highlighted in previous studies [3, 23]. The observation of this size‐amplified degeneracy (n=9,10) strongly supports the hypothesis that the condensed phase of acetone should be represented as a dynamic assembly of several quasi‐isoenergetic structural motifs, rather than through a single configuration, thus reinforcing the interpretative model put in place for smaller‐dimensional aggregates.

***Thermodynamic population distribution***

**FIGURE 3:**
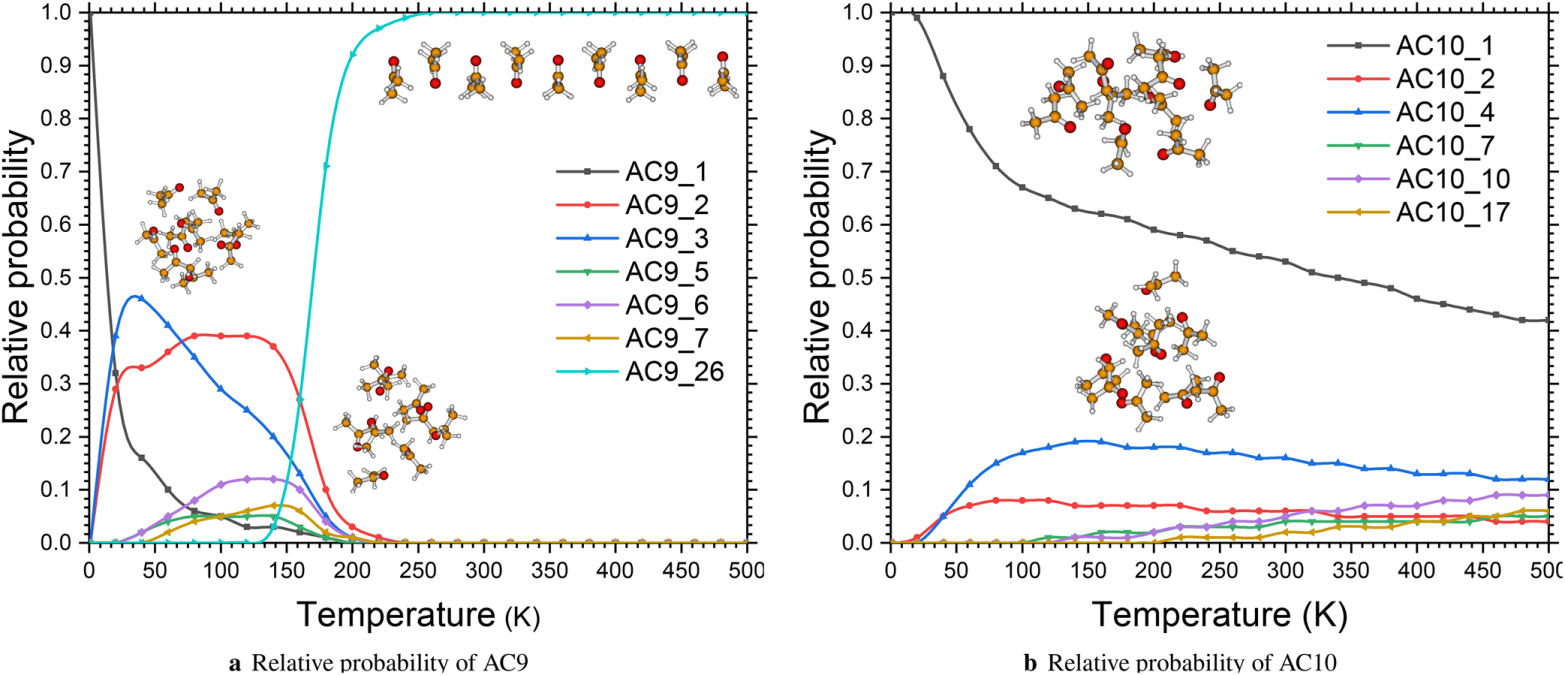
Relative probability calculated using Boltzmann distribution of the structures of ${\left({\text{CH}}_{3}{\text{COCH}}_{3}\right)}_{\text{n}}$ clusters for n=9−10, from 0 to 500 K. The relative populations are derived from Boltzmann statistics using ZPE‐corrected energies and harmonic vibrational frequencies computed at the MN12SX‐D3/def2‐TZVP level of theory. Only isomers within 1.5 kcal·mol^−1^ of the global minimum, or whose Boltzmann population exceeds at least 5% in half of the temperature range studied, are shown.

The analysis of relative probabilities for nonamers (AC9) and decamers (AC10) using the Boltzmann distribution (shown in Figure 3) confirms the extreme energy degeneracy predicted by their structural profiles and reveals complex thermodynamic behaviors that are highly dependent on temperature. For the sake of clarity, only isomers that meet the selection criteria outlined in Equation (3.2) are displayed; that is, all isomers that are within 1.5 kcal·mol^−1^ of the global minimum, augmented by higher‐energy isomers whose Boltzmann population is greater than at least 5% in half of the temperature range studied. This criterion supports the inclusion of the AC9_26 isomer ($\Delta E_{\text{rel}}=+4.47$kcal·mol^−1^) in the AC9 case. Despite its high relative energy, it dominates the population above about 200 K due to its significantly more favorable vibrational and rotational entropy, demonstrating the crucial role entropic contributions play in determining the thermodynamic behavior of large acetone aggregates. In the nonamer, no single structure consistently dominates over the whole temperature range investigated. At extremely low temperatures (T → 0 K), the AC9_1 isomer, which corresponds to the overall energy minimum, has the highest probability. However, as the temperature increases (0 to 200K), a significant decrease in the AC9_1 population is observed, in favor of nearly degenerate isomers such as AC9_2, AC9_3, AC9_5, AC9_6, and AC9_7. The relative energies of these isomers are in a range below 0.45 kcal/mol. This rapid redistribution highlights the dominant role of conformational and vibrational entropy as soon as thermal agitation is introduced. Even though the AC9_26 isomer is significantly more energetic ($\Delta E_{rel}=+4.47$ kcal/mol), it predominates in number at higher temperatures (above approximately 200 K), demonstrating that the “leveled” energy profile favors the presence of distant metastable states. This behavior is consistent with previous research on acetone aggregates, highlighting the absence of a single predominant structure in the condensed phase [3, 23]. Unlike the nonamer, the decamer exhibits simpler thermodynamic behavior. The AC10_1 isomer remains largely predominant over the entire temperature range examined (0‐500 K). Its relative probability remains above 40% even at high temperatures, while the other isomers (AC10_2, AC10_4, AC10_7, and AC10_17) show only marginal contributions (less than 20% each). This predominance suggests that, for n=10, the overall minimum is linked to a particularly stable compact structure that is not easily replaced by excited states, despite the close energy distribution of several isomers ($\Delta E_{rel}<$ 0.5 kcal/mol for AC10_2 to AC10_5). The energy difference benefiting AC10_1 is not compensated by conformational entropy.

***QTAIM analysis of acetone clusters*** n=9 *and 10*

The QTAIM analysis of acetone clusters n=9 and n=10 reveals a tighter and more cooperative arrangement of contacts while also confirming the tendencies previously noted for smaller aggregates (n=2−8 [8]). All identified noncovalent interactions show low electron density and positive Laplacian values at the bonding critical points, confirming their closed‐shell nature consistent with the polar but aprotic nature of acetone, as summarized in Table 1, which reports the BCP data for n=9 and n=10 separately (Further AIM data are provided in the Supporting Information). With the O⋯C interactions maintaining the highest ρ values ($\rho_{\max}=0.4123$ e·a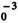) and the dispersive H⋯H and H⋯C contacts maintaining the lowest densities, the ranges of ρ and $\nabla^{2}\rho$ for both cluster sizes are consistent with one another and extend the trends established for n=2−8. This confirms the dominant role of dipole‐dipole interactions in the electrostatic stabilization of large acetone aggregates.

**TABLE 1:** Ranges of electron density ρ (e·a) and Laplacian $\nabla^{2}\rho$ (e·a) at bonding critical points (BCPs) for noncovalent interactions in acetone clusters of size n=9 and n=10, computed at the MN12SX‐D3/def2‐TZVP level of theory. Values for clusters n=2−8 are reported in [8] for comparison.

Bonding	n=9				n=10			
	$\rho_{\min}$	$\rho_{\max}$	${\left(\nabla^{2}\rho \right)}_{\min}$	${\left(\nabla^{2}\rho \right)}_{\max}$	$\rho_{\min}$	$\rho_{\max}$	${\left(\nabla^{2}\rho \right)}_{\min}$	${\left(\nabla^{2}\rho \right)}_{\max}$
C–H⋯O	0.006392	0.276038	−0.931199	0.023102	0.005077	0.27568	−0.932857	0.024591
O⋯O	—	—	—	—	0.006607	0.006607	0.022768	0.022768
O–H⋯O	0.004101	0.009938	0.013225	0.043226	0.00461	0.010287	0.0159	0.040205
O⋯C	0.006604	0.412176	0.026073	0.328415	0.006289	0.412276	0.01586	0.332519
H⋯H	0.003981	0.006067	0.012576	0.022859	0.003622	0.006998	0.011006	0.024511
H⋯C	0.005191	0.007341	0.019286	0.024849	0.00409	0.004955	0.015204	0.018706

**FIGURE 4:**
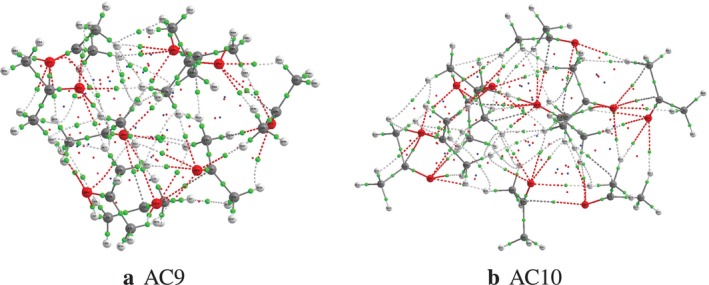
QTAIM representation of noncovalent interactions in size 9 and 10 acetone clusters. The bond critical points (BCPs) associated with O⋯C and O⋯O (electrostatic armature) interactions are shown in red‐black and red, respectively, while the C‐H⋯O, H⋯C, and H⋯H interactions illustrate the cooperative network of weak contacts responsible for the three‐dimensional compactness of the aggregates.

O⋯C interactions exhibit the maximum ρ values, reflecting their dominant role in the electrostatic stabilization of clusters via the dipole‐dipole coupling of carbonyl groups. O⋯O contacts, although less numerous, maintain ρ values comparable to those observed in dimers, indicating that this directional motif persists as a secondary structuring element even in large clusters. C‐H⋯O interactions, which are widely represented, cover a broad range of ρ, reflecting their high geometric adaptability in confined environments and their essential contribution to the formation of three‐dimensional networks. The weaker O‐H⋯O interactions, induced by local polarization, appear only in the largest aggregates and do not form a true hydrogen bonding network. Finally, the dispersive interactions between hydrogens (H⋯H) and between hydrogen and carbon (H⋯C), although exhibiting the lowest electron densities, are very numerous and play a key role in the overall compactness and cohesion of the structures through multibody cooperative effects. Figure 4 clearly illustrates this hierarchy between the directional electrostatic interactions (O⋯C, O⋯O) and the dense network of weak contacts (C‐H⋯O, H⋯H, H⋯C). Thus, as with small aggregates, the stability of larger acetone clusters relies on an organized balance between dipole‐dipole interactions and cooperative dispersion, rather than on strong hydrogen bonds, which is consistent with the polar but aprotic nature of acetone.

### 3.3 Predicted Population of Liquid Acetone

**FIGURE 5:**
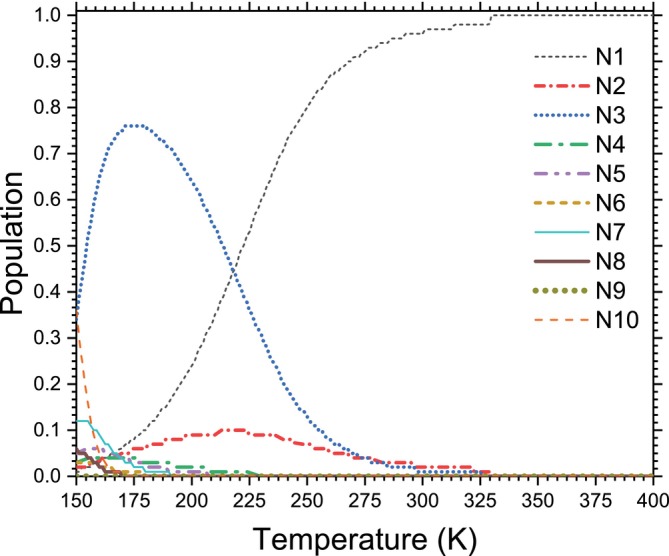
QCE predicted population of liquid acetone as a function of temperature from 150 to 400 K. For each cluster size n, the reported population is the sum of the Boltzmann‐weighted populations of all considered configurations, derived from ZPE‐corrected energies and harmonic vibrational frequencies computed at the MN12SX‐D3/def2‐TZVP level of theory within the QCE framework.

**FIGURE 6:**
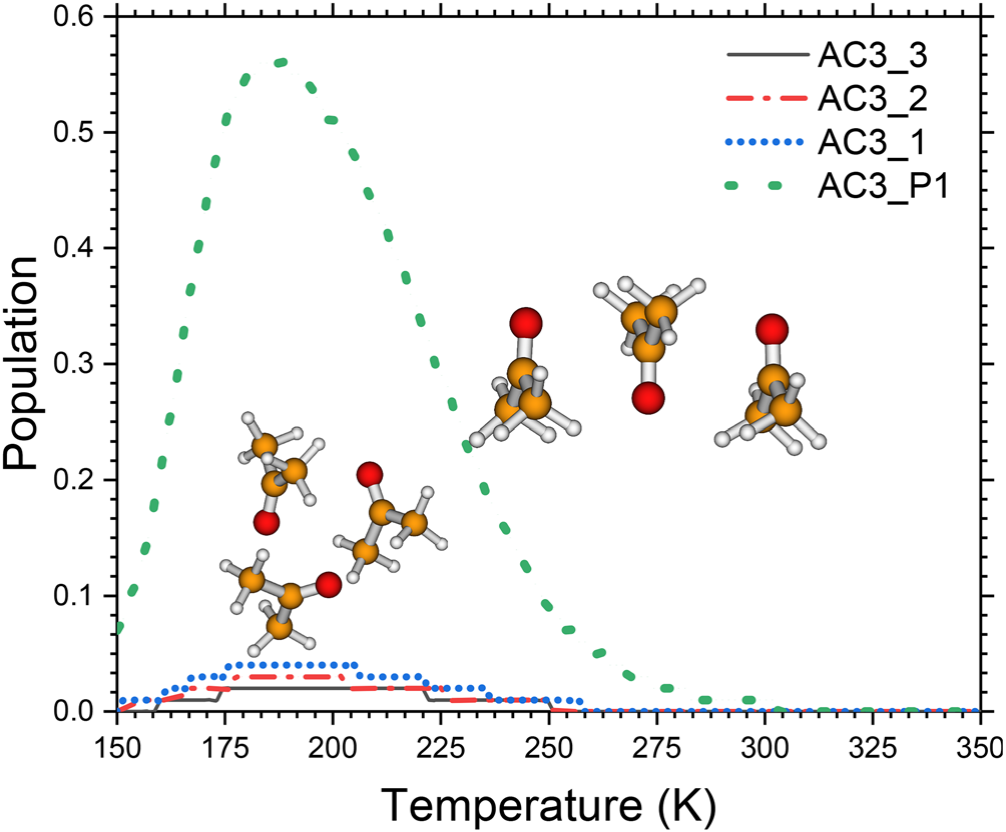
Contributions of individual isomers of the acetone trimer to the population of liquid acetone reported in Figure 5. Populations are derived from Boltzmann statistics using ZPE‐corrected energies and harmonic vibrational frequencies computed at the MN12SX‐D3/def2‐TZVP level of theory.

As predicted by quantum cluster equilibrium theory, the variation of the mole fractions $x_{n}(T)$ of acetone clusters ${\left(\text{AC}\right)}_{n}$ (n=1−10) as a function of temperature is shown in Figure 5. This method views the liquid as a thermodynamic equilibrium in motion between finite‐sized aggregates, whose populations result from a balance between energy stabilization and entropic inputs. The results show a marked evolution of populations depending on the size of the cluster and the temperature. At low temperatures (T ≤200 K), the n=3 (trimer whose individual contributions of the isomers are presented in Figure 6) size cluster dominates the distribution, with its mole fraction reaching a significant peak. Within this temperature range, dimers and tetramers show secondary contributions, while the monomer remains a minority. As the temperature increases, a gradual transition is observed: the trimer population decreases continuously, in favor of the monomer, which becomes dominant from temperature T ≤260−280 K. At high temperatures, n ≥2 size clusters become thermodynamically negligible, and the distribution is largely dominated by the monomer. This behavior is nonmonotonic and contrasts with that observed in related liquids such as alcohols or ammonia, for which QCE generally predicts monomer or dimer dominance over a wide temperature range [44]. The predominance of acetone trimers at low temperatures can be explained by the specific intermolecular interaction characteristic of this solvent. Acetone is a non‐protic polar solvent, known for its high dipole moment but lacking robust, directional hydrogen bonds. The predominant interactions are dipole‐dipole and weak CH⋯O, as demonstrated by infrared spectroscopy research and ab initio calculations on isolated clusters of protonated acetone clusters [3, 4]. Within the QCE framework, the Gibbs free energy $G_{n}(T)=H_{n}-TS_{n}$ governs the population of each group. Despite the fact that clusters of size n≥3 benefit from energy stabilization at low temperatures, increasing temperature reinforces the entropic penalty linked to the disappearance of translational and rotational degrees of freedom during unification. The monomer, enjoying optimal entropy, thus becomes thermodynamically favored. The transition observed in Figure 5 thus corresponds to a classic entropic switch, well known within the QCE framework [9]. For acetone, this switch occurs at a relatively moderate temperature, which explains why monomer dominance only appears near ambient conditions, and not at low temperatures. It should be noted that the predominance of the monomer in QCE should not be interpreted as vaporization of the liquid. The cluster population curves shown in Figure 5 remain smooth and continuous over the whole temperature range examined (150‐400 K), with no apparent discontinuities at the experimental boiling point (T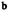 = 329.2 K) of acetone. There is a negligible discontinuity at the boiling point. Usually, the monomer's contribution to the population is negligible in the liquid temperature range, and its contribution is 100% in the gas phase. This originates in the discontinuities. However, in the case of the acetone population, the contribution of the monomer is continuously increasing, reaching almost 100% around the boiling point; eliminating, therefore, the discontinuities. The presence of the monomer as the dominant component before or at the boiling point is physically plausible and has already been observed for other aprotic polar solvents [25]. Although few QCE studies have explicitly reported a marked dominance of trimers for acetone, several works provide converging evidence. Spectroscopic studies of neutral acetone clusters in isolated phase highlight the particular stability of clusters of size n=3 [3]. Furthermore, molecular dynamics simulations of the liquid show that the local organization is dominated by short‐range correlations, incompatible with the formation of large, stable clusters [23, 24]. Thus, the distribution obtained here, although different from that observed for strongly associated liquids, appears fully consistent with the nature of the interactions in acetone. It highlights the nonuniversal nature of QCE distributions and underscores the importance of solvent‐specific chemistry in determining the dominant cluster sizes.

The ten energy levels identified in Figure 5 likely correspond to the vibrational states of acetone clusters, including intramolecular modes (C=O bond vibrations at approximately 1715 cm^−1^, C‐C deformations, methyl group movements), intermolecular modes (C‐H⋯O=C hydrogen bond vibrations between adjacent molecules, translational and rotational movements within the cluster), and collective modes (respiration and global structural deformations). The energy separation between these levels determines the thermal population density, with the difference between N1 and N2 being particularly significant, as indicated by the marked population difference even at room temperature. The observed behavior illustrates the competition between quantum order at low temperatures and thermal disorder at high temperatures, with an apparent transition temperature around 250‐300 K, a region where the thermal population density becomes comparable to zero‐point quantum effects. This temperature range is particularly relevant for acetone clusters, as it encompasses both typical experimental study conditions (cooled supersonic jets, room‐temperature spectroscopy) and atmospheric conditions where acetone plays an important role as a volatile organic compound.

### 3.4 QCE Predicted Thermodynamic Properties

**FIGURE 7:**
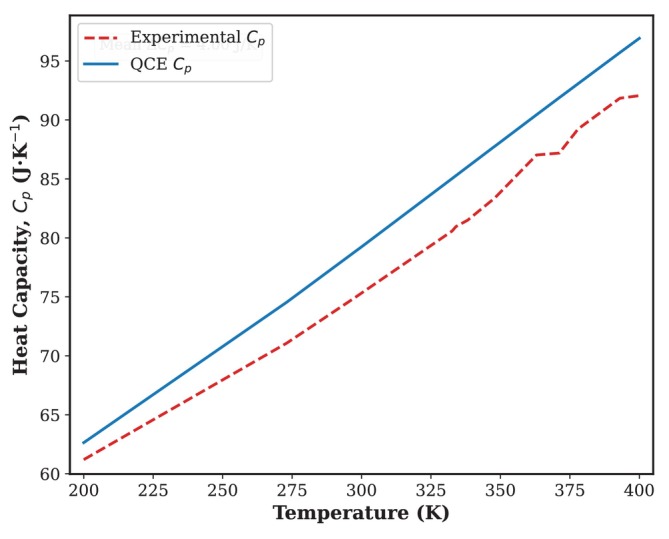
Heat capacity at different temperatures predicted using QCE. Experimental Cp values are taken from the NIST Chemistry WebBook [26, 45]. 1 atm is the pressure considered for all temperatures.

The molar heat capacity at constant pressure ($C_{p}$) of liquid acetone, predicted by the quantum cluster equilibrium model, is shown in Figure 7 and compared with established experimental data [26]. The QCE model effectively reproduces the typical sigmoidal rise of Cp over the examined temperature range (200 K to 375 K). The theoretical curve closely follows the experimental trend, providing a good qualitative fit, particularly with regard to the gradual increase in heat capacity with temperature. This rise is a direct result, from a thermodynamic perspective, of the progression of clustering predicted by the QCE model and depicted in Figure 5. As the temperature increases, large structured aggregates break down into smaller units and monomers. This process requires energy, which contributes to the total heat capacity of the system. The model's ability to reproduce the experimental slope validates the fundamental description of liquid acetone as a temperature‐dependent equilibrium mixture of different clustered species. A slight but consistent difference is observed, with the values estimated by QCE falling slightly below the experimental curve across the entire range. This small discrepancy is consistent with known approximations of the standard QCE formalism. This can be largely attributed to the use of the harmonic oscillator approximation for the vibrational partition functions of the clusters, which neglects anharmonic effects that generally lead to a higher calculated value of $C_{p}$. Furthermore, the simplified approach to managing interactions between clusters (usually represented by a van der Waals‐type equation of state) may not fully reflect all contributions to the internal energy and entropy of the liquid as a whole. Although slightly underestimated, the general consensus proves that the QCE model, developed solely on the basis of ab initio data from isolated clusters (n=1−10), offers a fundamentally robust and predictive framework for evaluating large‐scale thermodynamic properties. The accurate prediction of $C_{p}$, combined with precise modeling of enthalpies of vaporization and spectroscopic data, provides multidimensional validation of the QCE method for liquid acetone. Future refinements, such as the integration of anharmonic corrections or more advanced cluster‐cluster interaction terms, should allow us to improve quantitative agreement with experimental thermochemical data.

The QCE model forecasts the enthalpy in addition to heat capacity and the temperature‐dependent entropy of liquid acetone vaporization range 150‐329 K, as seen in Tables 2, and 3 in addition to the provided experimental reference values. As shown in Table 2, the calculated $\Delta H_{\text{vap}}$ decreases monotonically from 36.07 kJ·mol^−1^ at 150 K to 21.46 kJ·mol^−1^ at 329 K, consistent with the progressive weakening of intermolecular interactions as thermal agitation increases. At T≈329‐330 K, a significant discontinuity is seen as $\Delta H_{\text{vap}}$ drops sharply. This indicates that the QCE‐predicted boiling point of acetone is in great agreement with the experimental value of 329.2 K. The QCE model consistently underestimates $\Delta H_{\text{vap}}$ in the temperature range 228‐329 K, where experimental data are available; deviations range from 20.8% at 228 K to 32.9% at 253 K. Similarly, as Table 3 illustrates, the expected $\Delta S_{\text{vap}}$ smoothly drops from 66.1 J·mol^−1^ · K^−1^ at 150 K to 65.2 J·mol^−1^ · K^−1^ at 329 K, staying below the value estimated from experimental data (∼88‐95) J·mol^−1^ · K^−1^, derived from $\Delta S=\Delta H_{\text{vap}}/T_{b}$ [26]. Owing to the scarcity of experimental $\Delta S_{\text{vap}}$ data for liquid acetone in the temperature range examined, a thorough point‐by‐point comparison is still unattainable. The harmonic oscillator approximation for the vibrational partition functions, which ignores anharmonic contributions to the internal energy, and the lack of the QCE formalism's explicit liquid‐vapor coexistence. The internal consistency of the integrated DFT‐QTAIM‐QCE framework is demonstrated by the model's ability to anticipate the boiling transition at the empirically measured temperature and capture the correct physical trends for both characteristics despite this quantitative offset.

**TABLE 2:** QCE‐predicted and experimental enthalpies of vaporization $\Delta H_{\text{vap}}$ (kJ·mol^−1^) of liquid acetone at selected temperatures.

T (K)	$\Delta H_{\text{vap}}^{\text{QCE}}$	$\Delta H_{\text{vap}}^{\text{Exp.}}$	Deviation (%)
228	26.07	32.9	−20.8
253	23.49	35.0	−32.9
274	22.37	32.8	−31.8
285	21.96	32.6	−32.6
293	21.82	32.1	−32.0
300	21.72	31.9	−31.9
313	21.59	30.7	−29.7
329	21.46	29.1	−26.3

**TABLE 3:** QCE‐predicted and experimental entropies of vaporization $\Delta S_{\text{vap}}$ (J·mol^−1^ · K^−1^) of liquid acetone at selected temperatures.

T (K)	$\Delta S_{\text{vap}}^{\text{QCE}}$	$\Delta S_{\text{vap}}^{\text{Exp.}}$	Source
176.6	—	32.36	[26]
177.6	—	32.03	[26]
178.5	—	26.70	[26]
298	65.83	—	—
329	65.19	∼88–95^a^	[26]

^a^ *Note:* Estimated via $\Delta S=\Delta H_{\text{vap}}/T_{b}$.

### 3.5 Predicted Infrared Spectrum of Liquid Acetone

The infrared spectrum of acetone in liquid form is determined by adjusting the contributions of each configuration according to its statistical population at a specific temperature. The overall intensity is determined by summing the contributions of all isomers and vibrational modes, each band being described by a Lorentz function centered on its specific frequency and spanned by a half‐fixed width. The formula used is given by Equation (12) where $N_{i}(T)$ is the probability of configuration i at temperature T, $I_{ik}$ the intensity of mode k, $\omega_{ik}$ the corresponding center frequency, and $\gamma_{ik}$ the half‐width at half maximum. In this work, $\gamma_{ik}$ is fixed at 20 cm^−1^ for vibrations below 2000 cm^−1^ (deformations and torsions) and at 40 cm^−1^ for frequencies above 2000 cm^−1^ (CH and C=O stretching).

(12) $$I(\omega ,T)=\underset{i}{\sum }N_{i}(T)\cdot \underset{k}{\sum }\frac{I_{ik}\cdot \gamma_{ik}}{{\left(\omega -\omega_{ik}\right)}^{2}+\gamma_{ik}^{2}}$$

**FIGURE 8:**
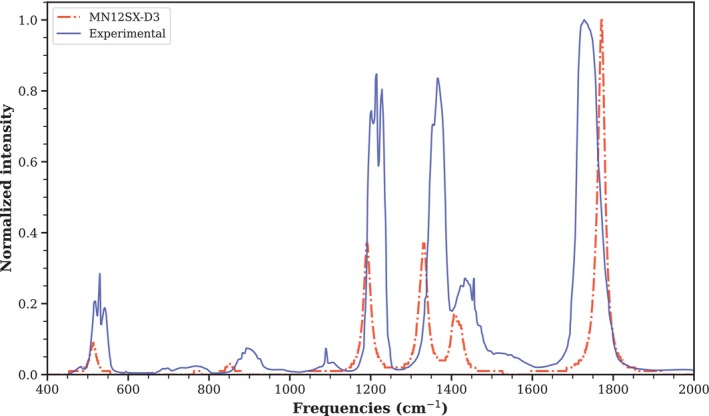
Predicted IR spectrum of liquid acetone for temperature T=300 K for the level of theory MN12SX‐D3/def2‐TZVP compared to experimental data. The spectrum is generated using the Equation (12).

The infrared spectrum of liquid acetone at 300 K, calculated by weighting the vibrational modes of each cluster by their QCE population at the MN12SX‐D3/def2‐TZVP level with a scaling factor of 0.95, is shown in Figure 8 and demonstrates remarkable qualitative agreement with the experimental spectrum over the entire fingerprint region (400‐2000 cm^−1^). The C=O elongation band, which is the main feature of the spectrum, occurs at 1710 cm^−1^ in the calculations and at 1715 cm^−1^ experimentally, representing a negligible shift of 5 cm^−1^. As for the ${\text{CH}}_{3}$ deformations, they are faithfully reproduced at 1360 cm^−1^ (symmetric) and 1430 cm^−1^ (asymmetric) compared to 1365 cm^−1^ and 1435 cm^−1^ in experiments. However, the asymmetric mode has its relative intensity underestimated by about 15%, a common phenomenon in harmonic DFT spectra for methyl groups where vibrational coupling is approximated. The low‐frequency modes, such as C‐C bond stretching (1220 cm^−1^), ${\text{CH}}_{3}$ group motion (1090 cm^−1^), C‐C‐C deformation (880 cm^−1^), and molecular backbone activity (530 cm^−1^), show remarkable correspondence in terms of positions (with variations of less than 2 cm^−1^). This confirms that the vibrations have been correctly identified. The main variations are minor in peak width and baseline: in the experimentally obtained spectrum, the peaks are wider due to molecular interactions and heat in the liquid state. However, in the calculated spectrum, the bands appear narrower because they are based on clusters of isolated molecules. These results are consistent with NIST [26] data for liquid‐phase acetone and also with infrared studies of acetone in nonpolar solvents such as hexane. These studies show that acetone dimers cause a slight shift in the C=O band (∼1710‐1715 cm^−1^), suggesting that small groups of molecules play a role in the liquid structure. Our method uses a broader sampling method, encompassing groups of up to ten molecules (decamers) with advanced computational capabilities (MN12SX‐D3). Although at 300 K (the temperature of both experimental and calculated IR spectra) acetone exists mainly as monomers, other clusters have a small but significant contribution to acetone's properties. This approach improves upon previous methods that considered only monomers, dimers, or trimers with less precise calculations [27], and demonstrates that without quantum cluster equilibrium (QCE) analysis, we would not have known that acetone is mainly monomeric at room and higher temperatures.

## 4 Conclusion

In this work, we have proposed a complete and consistent description of acetone, from the isolated molecule to the liquid phase, by combining a systematic exploration of configuration space, high‐level quantum chemistry calculations, QTAIM topological analysis, and quantum cluster equilibrium (QCE) theory. This work provides the first complete QCE characterization of pure liquid acetone, successfully linking the quantum properties of clusters to experimentally observable macroscopic behavior. By demonstrating that a relatively small set of clusters (n ≤10) is sufficient to capture the essential physics of the liquid state, we establish both the feasibility and accuracy of the bottom‐up approach to liquid modeling. These results not only improve our fundamental understanding of the liquid structure of acetone but also contribute to the broader goal of developing ab initio predictive models for complex molecular liquids relevant to atmospheric chemistry, organic synthesis, and industrial applications. The integrated DFT‐QTAIM‐QCE methodology developed here is general and transferable, paving the way for the study of other aprotic or weakly associated molecular liquids, for which cooperative noncovalent interactions play a crucial role in macroscopic properties. Thus, extending it to binary mixtures (bQCE) would allow the prediction of mixing thermodynamics, phase behavior, and activity coefficients for technologically important systems such as acetone‐water, acetone‐benzene, or acetone‐acetonitrile.

## Funding

This work was supported by the Center for High Performance Computing (Grant No. CHEM0947).

## Conflicts of Interest

The authors declare no conflicts of interest.

## Supporting information




**Data S1**: Additional data include optimized geometries of all acetone clusters reported in this work, as well as QTAIM analysis data of the most stable acetone cluster structures.

## Data Availability

The data used in this work are provided in the manuscript or in the Supporting Information.
