# Supplementary figures and images for: Comprehensive Modeling of Acetone Clusters: QTAIM Analysis and QCE Study
Source: J Comput Chem. 2026 Apr 20;47:e70380. doi: 10.1002/jcc.70380 (PMC13094332; doi:10.1002/jcc.70380)

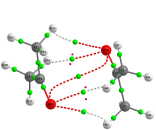

**a** AC2

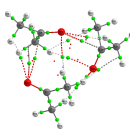

**b** AC3

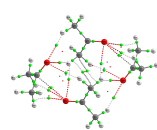

**c** AC4

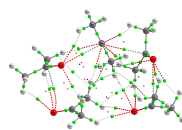

**d** AC5

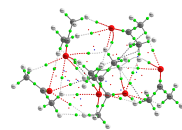

**e** AC6

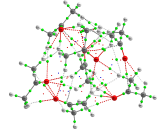

**f** AC7

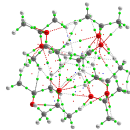

**g** AC8

Supplement: Supplementary file 1 — Data S1: Additional data include optimized geometries of all acetone clusters reported in this work, as well as QTAIM analysis data of the most stable acetone cluster structures. [file JCC-47-0-s001.zip › Supporting_information/Fig_QTAIM.pdf]

Statistical descriptors (kcal/mol)

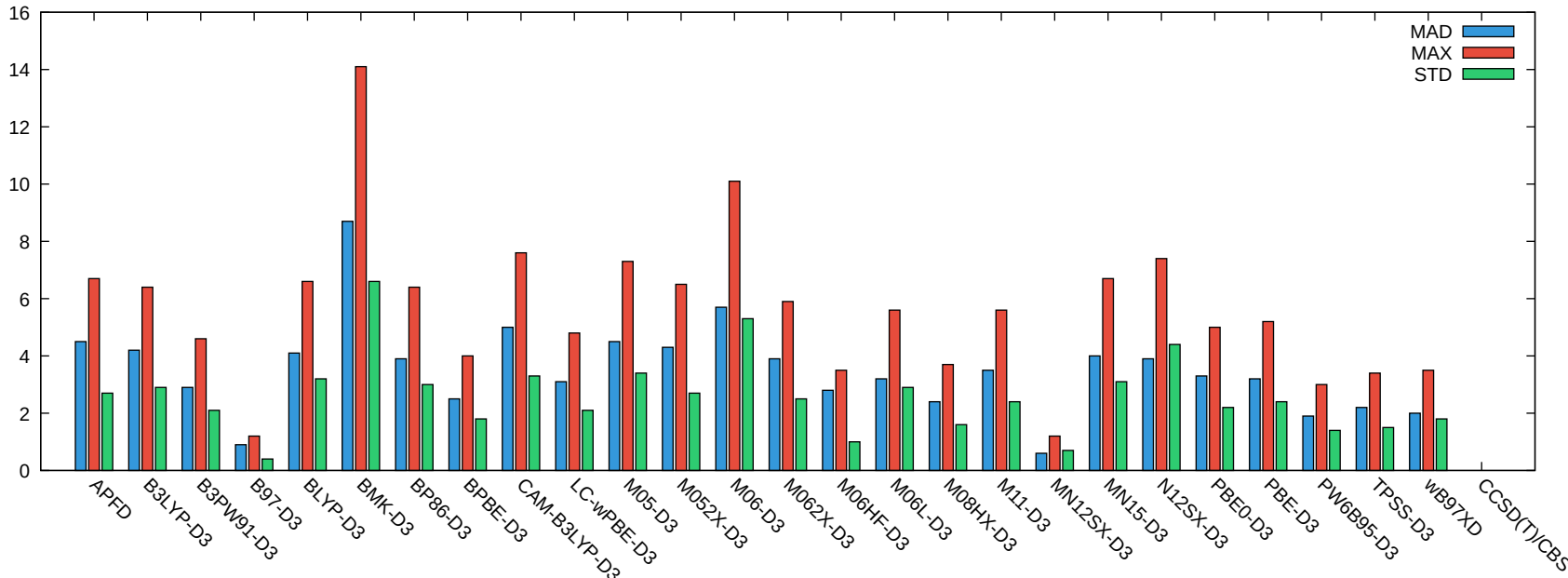

Supplement: Supplementary file 1 — Data S1: Additional data include optimized geometries of all acetone clusters reported in this work, as well as QTAIM analysis data of the most stable acetone cluster structures. [file JCC-47-0-s001.zip › Supporting_information/statistical_descriptors.pdf]
